# Monitoring response to a clinically relevant IDH inhibitor in glioma—Hyperpolarized ^13^C magnetic resonance spectroscopy approaches

**DOI:** 10.1093/noajnl/vdad143

**Published:** 2023-11-02

**Authors:** Donghyun Hong, Yaewon Kim, Chandrasekhar Mushti, Noriaki Minami, Jing Wu, Murali Krishna Cherukuri, Rolf E Swenson, Daniel B Vigneron, Sabrina M Ronen

**Affiliations:** Department of Radiology and Biomedical Imaging, University of California San Francisco, San Francisco, California, USA; Department of Radiology and Biomedical Imaging, University of California San Francisco, San Francisco, California, USA; National Cancer Institute, NIH, Bethesda, Maryland, USA; Department of Radiology and Biomedical Imaging, University of California San Francisco, San Francisco, California, USA; National Cancer Institute, NIH, Bethesda, Maryland, USA; National Cancer Institute, NIH, Bethesda, Maryland, USA; National Heart, Lung, and Blood Institute, NIH, Bethesda, Maryland, USA; Department of Radiology and Biomedical Imaging, University of California San Francisco, San Francisco, California, USA; Brain Tumor Research Center, UCSF, San Francisco, California, USA; Department of Radiology and Biomedical Imaging, University of California San Francisco, San Francisco, California, USA; Brain Tumor Research Center, UCSF, San Francisco, California, USA

**Keywords:** hyperpolarized 13C MRS, IDH inhibitor, imaging biomarker, mutant IDH glioma, therapeutic response

## Abstract

**Background:**

Mutant isocitrate dehydrogenase (IDHmut) catalyzes 2-hydroxyglutarate (2HG) production and is considered a therapeutic target for IDHmut tumors. However, response is mostly associated with inhibition of tumor growth. Response assessment via anatomic imaging is therefore challenging. Our goal was to directly detect IDHmut inhibition using a new hyperpolarized (HP) ^13^C magnetic resonance spectroscopy-based approach to noninvasively assess α-ketoglutarate (αKG) metabolism to 2HG and glutamate.

**Methods:**

We studied IDHmut-expressing normal human astrocyte (NHAIDH1mut) cells and rats with BT257 tumors, and assessed response to the IDHmut inhibitor BAY-1436032 (*n* ≥ 4). We developed a new ^13^C Echo Planar Spectroscopic Imaging sequence with an optimized RF pulse to monitor the fate of HP [1-^13^C]αKG and [5-^12^C,1-^13^C]αKG with a 2.5 × 2.5 × 8 mm^3^ spatial resolution.

**Results:**

Cell studies confirmed that BAY-1436032-treatment leads to a drop in HP 2HG and an increase in HP glutamate detectable with both HP substrates. Data using HP [5-^12^C,1-^13^C]αKG also demonstrated that its conversion to 2HG is detectable without the proximal 1.1% natural abundance [5-^13^C]αKG signal. In vivo studies showed that glutamate is produced in normal brains but no 2HG is detectable. In tumor-bearing rats, we detected the production of both 2HG and glutamate, and BAY-1436032-treatment led to a drop in 2HG and an increase in glutamate. Using HP [5-^12^C,1-^13^C]αKG we detected metabolism with an signal-to-noise ratio of 23 for 2HG and 17 for glutamate.

**Conclusions:**

Our findings point to the clinical potential of HP αKG, which recently received FDA investigational new drug approval for research, for noninvasive localized imaging of IDHmut status.

**Key Points**

- A new hyperpolarized substrate combined with a novel acquisition method improves the imaging of 2HG production and its modulation with treatment.
- Hyperpolarized MR spectroscopy-based metabolic imaging can assess response to IDH inhibitors.

**Importance of the Study**

Mutant isocitrate dehydrogenase (IDH) catalyzes the conversion of α-ketoglutarate (αKG) to 2-hydroxyglutarate (2HG), which is responsible for driving tumor development. Inhibition of 2HG production is being evaluated as a potential therapy and shows enhanced survival. However response is typically associated with inhibition of tumor growth, which can be challenging to assess. This study demonstrated the utility of a novel ^13^C magnetic resonance spectroscopy (MRS) pulse sequence combined with 2 hyperpolarized (HP) agents, the commercially available [1-^13^C]αKG and a recently synthesized [5-^12^C,1-^13^C]αKG, for acquiring high-resolution MR spectroscopic imaging data that can be used to monitor 2HG synthesis as well as its inhibition with clinically relevant mutant IDH inhibitors. This study demonstrates the value of HP ^13^C MRS as an important noninvasive imaging tool for monitoring drug delivery and therapeutic action within the tumor, thus providing the oncologist with a valuable tool to make data-driven decisions for mutant IDH inhibitor-treated patients.

Whereas historically brain tumors were classified based on their histological presentation, the recent 2021 WHO classification of glioma is based on molecular events.^1,2^ In this context, mutations in the enzyme isocitrate dehydrogenase (IDH1 or IDH2) are the first molecular events that now characterize adult lower-grade gliomas, which are comprised of grades 2 and 3 oligodendrogliomas and astrocytomas,^1,3^ as well as grade 4 astrocytomas.^1^ Mutant IDH catalyzes the conversion of α-ketoglutarate (αKG) to the oncometabolite 2-hydroxyglutarate (2HG), which is then responsible for driving tumor development via alterations in genome-wide histone and DNA methylation patterns.^4–6^ Since 2HG plays a key role in tumor development, inhibition of its production via mutant IDH targeting has been considered a potential therapeutic strategy, either alone or in combination with other anticancer drugs. Importantly, clinical trials of mutant IDH inhibitors have shown enhanced patient survival, but in most cases, this is associated with inhibition of tumor growth rather than tumor shrinkage.^7–9^ As a result, pretreatment information regarding tumor growth rate is required in order to assess the impact of treatment, and confirming drug action at the tumor site can be challenging. This challenge can be addressed by directly monitoring the production of 2HG by mutant IDH. Noninvasive in vivo imaging of 2HG production could therefore improve the characterization of brain tumors and the monitoring of their response to mutant IDH inhibitors.

Magnetic resonance spectroscopy (MRS) is a translational noninvasive method for monitoring localized tumor metabolism. ^1^H MRS, which can be readily implemented on most clinical magnetic resonance imaging (MRI) scanners, can be used to monitor steady-state metabolite levels. It has been used to detect the elevated levels of 2HG present in mutant IDH gliomas in patients,^10–13^ animal models,^14^ and cells.^15^ It has also been used to identify the broader metabolic reprogramming that occurs as a result of the IDH mutation and 2HG accumulation.^15,16^ A complementary metabolic imaging method is hyperpolarized (HP) ^13^C spectroscopy in combination with the dissolution dynamic nuclear polarization (d-DNP) technique,^17^ which highly enhances the signal-to-noise ratio (SNR) of ^13^C-labeled compounds. The enhanced SNR has made it possible to monitor the fate of labeled metabolites in vivo, providing a noninvasive method to evaluate real-time metabolic fluxes. Importantly, this method has been applied not only in cells and animal models but also in vivo in patients with multiple tumor types, including mutant as well as wild-type IDH glioma tumors^18–21^

In the context of mutant IDH gliomas, we have previously shown that HP [1-^13^C]αKG can be used to monitor both mutant IDH-driven 2HG production and normal αKG conversion to glutamate.^22–24^ We have also shown that in cells we can use ^1^H MRS to detect a drop in 2HG and an increase in glutamate following treatment with the clinically relevant IDH inhibitor AG-881. This was accompanied by an HP ^13^C MRS-detectable drop in flux from HP αKG to 2HG, and an increase in flux from αKG to glutamate.^25^ Subsequent in vivo studies from our lab confirmed the simultaneous, and opposite, modulation of ^1^H MRS-detectable 2HG and glutamate in rodent models with patient-derived mutant IDH tumors treated with both AG-881 and BAY-1436032.^14^ These ^1^H MRS-detectable metabolic changes were also consistent with findings in treated patients.^26^

However, to date, no studies have reported on using HP ^13^C MRS with [1-^13^C]αKG to monitor response to mutant IDH inhibitors in the in vivo setting. The main reason for this is illustrated in our prior work^22–24^ wherein we showed that the proximity of the HP 2HG peak (183.9 ppm) to the 1.1% natural abundance [5-^13^C]αKG peak (184 ppm) results in the detection of a single composite peak in vivo. Furthermore, early pulse sequences resulted in a somewhat limited SNR of 1.4 for 2HG.^23^ Accordingly, monitoring 2HG levels, or their modulation with treatment, has required careful postprocessing and analysis.^22–24^ Recently however, the synthesis of [5-^12^C,1-^13^C]αKG, and its utility as a substrate that allows for the detection of the [1-^13^C]2HG peak without interference from the natural abundance [5-^13^C] αKG peak, were demonstrated.^27^ The goal of this study was therefore to assess the utility of both the commercially available [1-^13^C]αKG and the recently synthesized [5-^12^C,1-^13^C]αKG, combined with an improved pulse sequence, to acquire high-resolution MR spectroscopic imaging data that can inform on the impact of mutant IDH inhibitors.

## Methods

### Cell and animal models

Normal Human Astrocytes (NHA) expressing mutant IDH R132H (NHAIDH1mut) were generated as previously described,^28^ and cultured in basal medium (Dulbecco’s Modified Eagle Medium; Gibco, USA) supplemented with 10% fetal calf serum, 2 mM of glutamine, and 100 U/ml of penicillin and streptomycin as previously.^15^ All animal studies were performed under UCSF Institutional Animal Care and Use Committee approval. Approximately 3 × 10^5^ BT257 patient-derived mutant IDH astrocytoma, courtesy of the Weiss lab (University of Calgary),^29^ were injected into the right hemisphere of the rat brain (Athymic male nu/nu rats; 4–5 week old; 150–180 g; Envigo Inc.) as previously described.^25^ Tumor growth was then monitored weekly using T_2_-weighted imaging. Imaging was performed using a preclinical MR scanner (3 T Biospin, Bruker) and a multi-slice spin-echo sequence with the following parameters: echo time (TE) = 64 ms, repetition time (TR) = 3484 ms, matrix = 256 × 256, resolution = 0.15 × 0.15 mm^2^, slice thickness = 1 mm, and the number of averages = 6. Tumor volume was measured with ImageJ software (ver. 1.53t, NIH, MD).

### Treatment

Cells and animals were treated with the brain-penetrant clinically relevant mutant IDH1 inhibitor BAY-1436032 (#HY-100020, MedChemExpress).

NHAIDH1mut cells were treated every 24 h for 3 days with the half-maximal inhibitory concentration (IC50) of BAY-1436032 or DMSO (vehicle, 0.2 %, MilliporeSigma) (*n* = 4 in each group). IC50 values were estimated based on enzyme activity assay and using a Tecan M200 Pro Infinity plate reader (Tecan Systems, Inc.) as described in our previous publication.^25^

For animal studies, when tumor volume reached ~27 mm^3^, we considered this point “day zero” (Day 0) and acquired both ^1^H and HP ^13^C MRS data (*n* = 6). After acquiring the Day 0 data set, we started treatment. Animals were injected intraperitoneally with a concentration of 150 mg/kg^14^ of BAY-1436032 every 24 h until their endpoint and ^1^H and HP ^13^C MRS data were acquired again on Day 10 (*n* = 4). A tumor control group was treated with vehicle (500 µl of DMSO plus 3 ml of saline), but because of their rapid decline, these animals were only investigated for tumor growth and survival (*n* = 5).

### ^1^H MRS

For cell studies, approximately 12 × 10^6^ treated or control cells were extracted using the dual-phase extraction methods.^25^^1^H spectroscopy was acquired on a 500 MHz NMR system (Varian) using 1D water presaturation ZGPR sequence, flip angle = 90°, TR = 3 s, 256 averages. The spectral baseline was then manually corrected, and metabolite concentrations were quantified using the area under the curve (peak) on MNOVA (ver. 12.0.4, Mestrelab Research). Metabolite concentrations were normalized to cell number and TSP as previously.^25^

In vivo, ^1^H single voxel spectroscopy data were acquired from pre (Day 0) and post (Day 10) treatment time points using the same 3 T preclinical MR system used for MRI and equipped with a quadrature ^1^H-^13^C volume coil (45 mm inner diameter, Neos-Biotech). A 4 × 4 × 4 mm^3^ isotropic voxel was fully located inside the tumor region and the PRESS (Point RESolved Spectroscopy) sequence^30^ was used to acquire data with the following parameters: TE = 16 ms (TE1 = TE2 = 8 ms), TR = 2500 ms, number of averages = 512, spectral resolution = 1024, spectral bandwidth = 10 ppm, and VAPOR water suppression.^31^ Spectra were quantified with LCModel (ver. 6.3-1, Provencher). A custom-made basis set that includes 2HG, alanine, aspartate, glycerophosphocholine, free choline, phosphocholine, creatine, phosphocreatine, γ-aminobutyric acid, glucose, glutamine, glutamate, glycine, glutathione, myoinositol, lactate, N-acetylaspartate, N-acetylaspartylglutamate, phosphoethanolamine, scyllo-inositol, and taurine was simulated using the NMRSIM module included in TOPSPIN suite (Version 3.6, Bruker) with identical parameters used for the in vivo acquisition (eg echo time, RF pulse timing, resonance frequency, and acquisition bandwidth). Chemical shift and J-coupling values for each metabolite were taken from a previous study.^32^ LCModel was then used to quantify the MRS-detectable metabolites of interest with the default control parameters LCModel provides.

### HP ^13^C MRS

[1-^13^C]αKG and [5-^12^C,1-^13^C]αKG were prepared as previously^24^ and polarized using a Hypersense polarizer. *T*_1_ values were also determined as previously.^24^

For cell studies, 500 μl of HP αKGs were injected into the supernatant of cell lysates from ~2.5 × 10^8^ cells as previously described.^25^ HP ^13^C spectra were acquired using a 500 MHz spectrometer (Agilent Technologies) using FA = 13°, TR = 3 s, and repetition = 100. Metabolite peaks were quantified with MNOVA and normalized to substrate and cell number.

**Figure 1.**
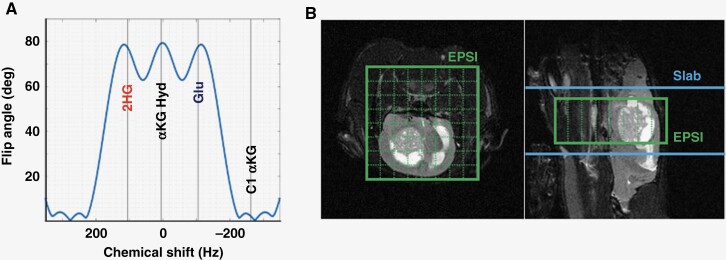
(A). Multiband excitation pulse newly designed for this study. (B). Illustration of matrix orientations for the slab (blue) and EPSI (green) acquisitions on typical T2-weighted axial (left) and sagittal (right) tumor images.

For animal studies, an improved multiband excitation pulse was designed as shown in Figure 1A. Our goal was to maximize the SNR of the product peak and conserve the magnetization of the substrate for subsequent metabolic conversion to the downstream products (eg 2HG) as long as possible. Our designed RF pulse has 4 different excitation bands with different flip angles (FA) as follows: 2HG_FA_ = 76°, αKG-Hyd_FA_ = 78°, glutamate _FA_ = 76°, and αKG_FA_ = 2° (the pulse was designed for a 0° flip angle for the αKG peak, however, an approximately 2° flip angle was applied in practice due to the ripple of the main RF band). The in-house software package available online (https://github.com/LarsonLab/hyperpolarized-mri-toolbox)^33^ in Matlab (ver. 2022b, Mathworks) designed RF pulses that were used for both the slab and ESPI acquisition sequences. Figure 1B shows the matrix orientations for the slab (blue) and EPSI (green) acquisitions on a typical T_2_-weighted axial (left) and sagittal (right) tumor image.

HP data were acquired from healthy animals, and tumor-bearing treated animals at pre (Day 0) and post (Day 10) treatment time points. Data were acquired immediately after injecting 2.5 ml of HP [1-^13^C]αKG through the tail vein over 12 s, and 2 h later data were acquired following injection of 2.5 ml of [5-^12^C,1-^13^C]αKG. Data were acquired using the same coil and scanner described above. We also confirmed that the order in which the substrates were injected did not have an impact on our results by performing a small-scale study on 2 animals comparing the 184-ppm peak when the order of substrate injection was reversed. Specifically, the normalized AUC of (C5 αKG + 2HG) at 184 ppm was 0.50 ± 0.12 (*n* = 6) when HP [1-^13^C]αKG was injected first, and it was 0.54 ± 0.04 (*n* = 2) when HP [1-^13^C]αKG was injected second. When [5-^12^C,1-^13^C]αKG was injected first the normalized AUC of 2HG at 184 ppm was 0.54 ± 0.10 (*n* = 2) and it was 0.59 ± 0.11 (*n* = 6) when [5-^12^C,1-^13^C]αKG was injected second.

The slab-localized method acquired a one-dimensional dynamic data set by positioning the slab axially to cover the whole tumor region. Acquisition parameters were slab thickness = 12 mm, data points = 1024, acquisition bandwidth = 300 ppm, temporal resolution = 3 s, and iterations = 24. The integrals of 2HG and glutamate were quantified at each time point and normalized to the αKG-hydrate peak to monitor metabolism over time. Overall changes were determined by quantifying the area under the curve (AUC) of this temporal evolution. The same animals were used to monitor the fate of both HP substrates. The SNR of each metabolite was measured as the ratio of the peak amplitude to the standard deviation of the noise.

MR spectroscopic imaging (MRSI) data were acquired using echo-planar spectroscopic imaging (EPSI)^34^ after injecting HP [5-^12^C,1-^13^C]αKG as above to pretreatment and posttreatment animals. Acquisition parameters for imaging were FOV = 20 × 20 × 8 mm^3^, matrix size = 8 × 8 × 1, spatial resolution = 2.5 × 2.5 × 8 mm^3^, TR = 4800 ms TE = 8 ms, temporal resolution = 3 s and iterations = 20. Flip angles were identical to the slab acquisition. Acquired spectra were denoised using tensor denoising^35–37^ and quantified with an in-house Matlab script (ver. 2021b, Mathwork).

### Statistics

All results are expressed as mean ± SD. An unpaired Student’s *t*-test was used to compare control and treatment groups and a paired Student’s *t*-test was used to compare the results obtained from the same animal when using the 2 HP substrates. A *P-value* ≤ .05 was considered statistically significant. *Signifies *P* < .05, **P < .01, ***P < .001, and ns = not significant.

## Results

Because we were investigating the utility of both [1-^13^C]αKG and [5-^12^C,1-^13^C]αKG, we first wanted to determine the T_1_ relaxation time of [5-^12^C,1-^13^C]αKG to confirm that it is comparable to the T_1_ of [1-^13^C]αKG. We determined that the T_1_ relaxation times of [1-^13^C]αKG and [5-^12^C,1-^13^C]αKG at 11.6 T were 24.3 ± 1.0 (*n* = 3) s and 25.6 ± 2.2 s (*n* = 3), respectively, confirming that the C1 resonances of the 2 αKG substrates have comparable relaxation times. When comparing our *T*_1_ values with those reported in the literature (Supplementary Table S1), our values were slightly longer than those of other studies likely due to somewhat different preparations of the agents for polarization, most notably the concertation of Gadolinium (0.3 mM in our study and 2.5 mM in other studies^27,38^). ^13^C spectra obtained for both HP agents are illustrated in Supplementary Figure S1 and confirmed that the [5-^12^C,1-^13^C]αKG, in contrast to [1-^13^C]αKG, does not show the [5-^13^C]αKG peak at 184 ppm.

**Figure 2.**
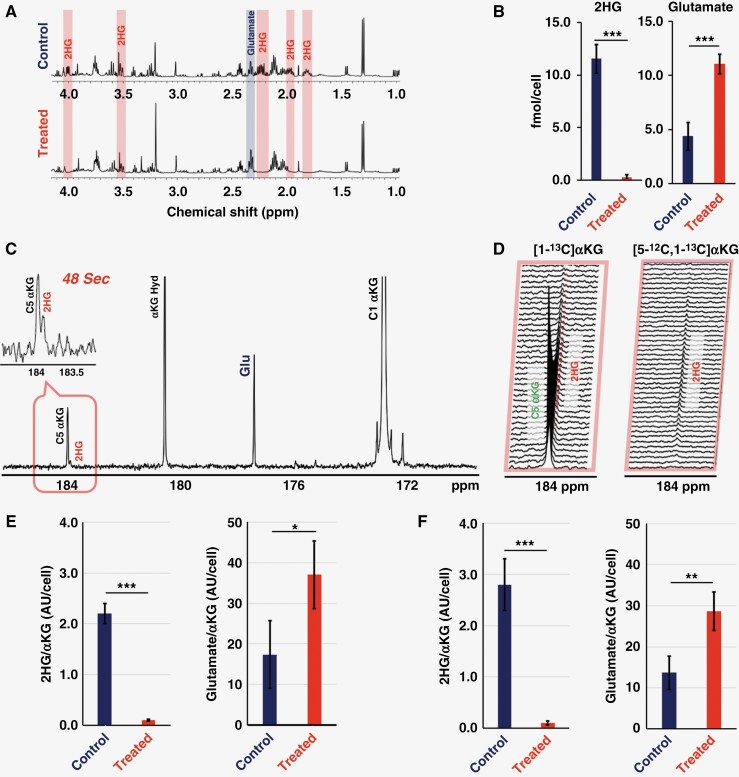
(A) ^1^H MRS spectra from control and treated NHAIDH1mut cells with 2HG and glutamate highlighted in red and blue, respectively. (B) Comparison of 2HG and glutamate levels prior to and following BAY-1436032 treatment as quantified from the ^1^H MRS spectra of control and treated NHAIDH1mut cells. (C) Summed ^13^C MRS spectrum over time post-HP [1-^13^C]αKG injection in control cells. Insert panel shows the spectrum at 48 s illustrating 2HG at 183.9 ppm and the natural abundance peak of [5-^13^C]αKG at 184 ppm. (D) Spectra around 184 ppm following injection of the 2 HP substrates: [1-^13^C]αKG (left) and [5-^12^C,1-^13^C]αKG (right) and illustrating 2HG production without the [5-^13^C]αKG peak for [5-^12^C,1-^13^C]αKG. (E) Quantification of HP 2HG and glutamate levels in control and treated cells following [1-^13^C]αKG injection. (F) Quantification of HP 2HG and glutamate levels in control and treated cells following [5-^12^C,1-^13^C]αKG injection. An unpaired Student’s *t*-test was used to compare the control (*n* = 4) and treatment (*n* = 4) groups. A *P*-value ≤ .05 was considered statistically significant. * signifies *P* < .05, **P < .01, ***P < 0.001, and ns = not significant.

Next, we wanted to confirm the utility of HP [5-^12^C,1-^13^C]αKG as an indicator of response to the mutant IDH inhibitor BAY-1436032 in NHAIDH1mut cells, which we had previously investigated using HP [1-^13^C]αKG.^25^ To determine the appropriate treatment dose, we assessed the effect of BAY-1436032 on mutant IDH enzyme activity in our cells (Supplementary Figure S2) and chose to treat with 0.5 µM BAY-1436032, the dose that dropped enzyme activity by half and similar to our previous study.^25^Figure 2A shows the ^1^H MRS data from NHAIDH1mut cells and demonstrates that similar to prior findings with other IDH inhibitors and models, the cells treated with BAY-1436032 showed the expected significant decrease in 2HG levels from 11.56 ± 1.36 to 0.27 ± 0.26 fmol/cell with *P-value* < .001 accompanied with a significant increase in glutamate from 4.60 ± 1.01 to 11.80 ± 2.24 fmol/cell with *P-value* < .001 (see Figure 2B).

Figure 2C shows an example ^13^C spectrum following injection of HP [1-^13^C]αKG into control cells and illustrates the proximity of the 2HG peak and [5-^13^C] αKG peaks within 0.1 ppm of each other. This is further illustrated in Figure 2D (left), which shows the dynamic production of downstream 2HG from HP [1-^13^C]αKG. When HP [5-^12^C,1-^13^C]αKG was used (Figure 2D (right)), dynamic 2HG production was also observed, but, as expected, without the proximal [5-^13^C] αKG peak. Following treatment with BAY-1436032, 2HG production dropped significantly from 2.20 ± 0.21 to 0.21 ± 0.02 AU/cell (*P-*value* *< .001) using HP [1-^13^C]αKG (Figure 2E) or from 2.80 ± 5.11 to 0.22 ± 0.10 AU/cell (*P-*value* *< .001) when using HP [5-^12^C,1-^13^C]αKG (Figure 2F). Findings using either HP substrate were comparable (*P-*value* *= .63). At the same time, glutamate levels increased significantly, and again data generated using HP [1-^13^C]αKG (from 17.63 ± 5.16 to 36.98 ± 11.61 AU/cell, *P-*value = .014, Figure 2E) or HP [5-^12^C,1-^13^C]αKG (from 11.96 ± 4.77 to 27.83 ± 4.16 AU/cell, *P-*value < .001, Figure 2F) were within experimental error (*P-*value = .61).

**Figure 3.**
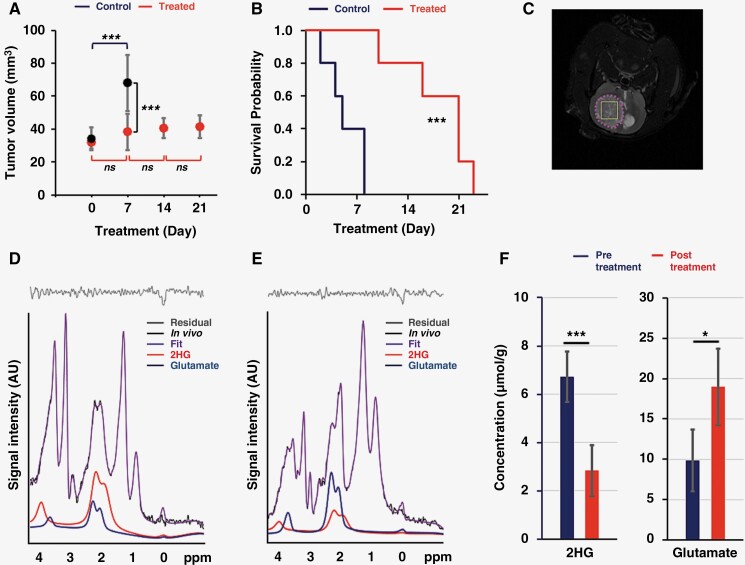
(A) Temporal evolution of average tumor volume in control and treated groups using an unpaired Student’s *t*-test for comparison of groups. (B) Kaplan–Meier survival probability of BT257 tumor-bearing rats comparing control and treated animals. (C) T_2_-weighted BT257 tumor image (pink) illustrating the single voxel tumor localization (yellow) for ^1^H spectroscopy. ^1^H single voxel spectra with LCModel fitting of 2HG and glutamate prior to (D) and following (E) treatment. (F) Comparison of 2HG and glutamate levels prior to (*n* = 6) and following (*n* = 4) treatment based on ^1^H spectra. Statistical significance was estimated with a paired *t*-test. A *P*-value ≤ .05 was considered statistically significant. *Signifies *P* < .05, **P < .01, ***P < .001, and ns = not significant.

In an effort to investigate more clinically relevant models, we next studied patient-derived BT257 mutant IDH1 astrocytoma tumors implanted in the rat brain.^14,24^ As demonstrated in Figure 3A, tumor volumes estimated by T_2_-weighted MRI showed that BAY-1436032 treatment significantly inhibited tumor growth in treated animals when compared to controls (*P-*value < .001). Associated with inhibition of tumor growth in Figure 3B, BAY-1436032 also increased the survival of treated animals, with the control group surviving up to 8 days following the onset of treatment, whereas the treated group survived up to 23 days (hazard ratio = 0.31, *P-*value = .0012).

Figure 3C illustrates the localization of the ^1^H spectroscopy single voxel on a T_2_-weighted image of a BT257 tumor and the ^1^H MRS results with their associated LCModel fit lines are illustrated in Figure 3D prior to treatment and in Figure 3E following treatment. The LCModel fit-based quantification (Figure 3F) showed that 2HG levels dropped significantly from 6.72 ± 1.04 to 2.83 ± 1.07 μmol/g (*P-value* < .001) and glutamate levels increased significantly from 9.84 ± 3.81 to 18.96 ± 4.72 μmol/g (*P-value *= .012), consistent with the observations in cells and similar to our prior observations of glioma models in mice.^14^

**Figure 4.**
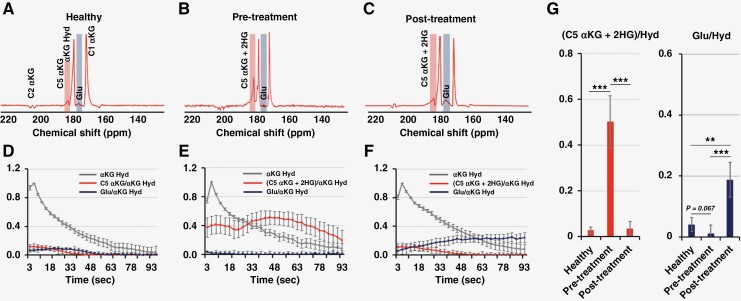
Summed ^13^C spectra of HP [1-^13^C]α-ketoglutarate metabolism over time in (A) a healthy rat and (B) pre- and (C) post-BAY-1436032 treatment BT257 tumor-bearing rats. Dynamic curves of each metabolite from corresponding models are shown in (D), (E), and (F). (G) Quantified AUC of the dynamic curves from (D, *n* = 5), (E, *n* = 6), and (F, *n* = 4) with comparisons using an unpaired *t*-test. A *P*-value ≤ .05 was considered statistically significant. *Signifies *P* < .05, **P < .01, ***P < .001, and ns = not significant.

We then proceeded to assess the effects of treatment using both our HP substrates. Figure 4A–C illustrates the HP sum spectra over time from healthy animal brains, pretreatment tumors, and posttreatment tumors, respectively, following injection of HP [1-^13^C]αKG. Figure 4D–F shows the time course of HP metabolite intensities and Figure 4G reflects the quantification of these metabolites. Spectra of healthy animals demonstrate the presence of glutamate production at 177 ppm. A small peak was also observed at 184 ppm with a similar time course to the [1-^13^C] αKG peak and likely reflecting [5-^13^C] αKG. Tumor animals before treatment demonstrate the presence of a clear, and relatively elevated, composite resonance of [1-^13^C]2HG and [5-^13^C]αKG at 184 ppm, and barely detectable glutamate. The time course of the peak at 184 ppm is consistent with our previous results^23,24^ with an apparently delayed build-up of signal reflecting 2HG production while [5-^13^C]αKG is decaying. Importantly, after treatment with BAY-1436032, the normalized peak at 184 ppm dropped significantly (from 0.50 ± 0.11 to 0.04 ± 0.03, *P-*value < .001) to a level comparable to that observed in the normal animals, reflecting the expected drop in 2HG production following treatment as observed in cells and consistent with the drop in total 2HG observed in the ^1^H spectrum. At the same time, the glutamate signal was significantly increased (from 0.01 ± 0.03 to 0.19 ± 0.06, *P-*value < .001) compared to the pretreated spectrum, again in line with our findings in cells and the increase in total glutamate levels observed by ^1^H MRS.

**Figure 5.**
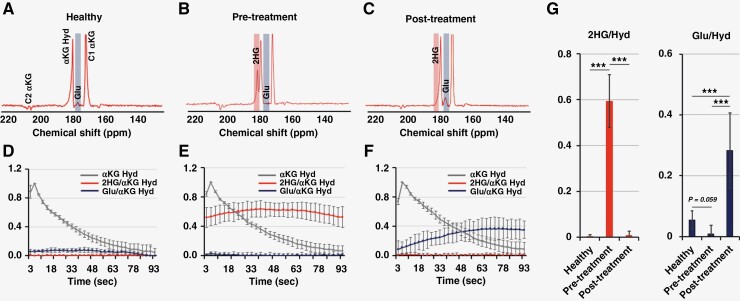
Summed spectra of HP [5-^12^C,1-^13^C]α-ketoglutarate metabolism over time in (A) a healthy rat and (B) pretreatment and (C) posttreatment BT257 tumor-bearing rats. Dynamic curves of each metabolite from corresponding models are shown in (D), (E), and (F). (G) Quantified AUC of dynamic curves from (D, *n* = 5), (E, *n* = 6), and (F, *n* = 4) with comparisons using an unpaired t-test. A *P*-value ≤ .05 was considered statistically significant. *Signifies *P* < .05, **P < .01, ***P < .001, and ns = not significant.

Figure 5 illustrates our findings in the same set of animals but using HP [5-^12^C,1-^13^C]αKG. Importantly, and in line with the data in cells, we do not detect a peak at 184 ppm in normal animals and the time course for 2HG production in the pretreatment animals shows a rapid and monotonic increase, without the intermediate decay observed with HP [1-^13^C]αKG but with a maximum observed around 39–48 s, comparable to the data with [1-^13^C]αKG. Furthermore, the quantification of the HP data is consistent with data in cells and with [1-^13^C]αKG showing a clear and significant drop in 2HG production (from 0.59 ± 0.11 to 0.01 ± 0.02, *P-*value < .001) and a clear and significant increase in glutamate production (from 0.01 ± 0.03 to 0.28 ± 0.12, *P-*value < .001) from αKG following treatment with the mutant IDH inhibitor BAY-1436032.

**Figure 6.**
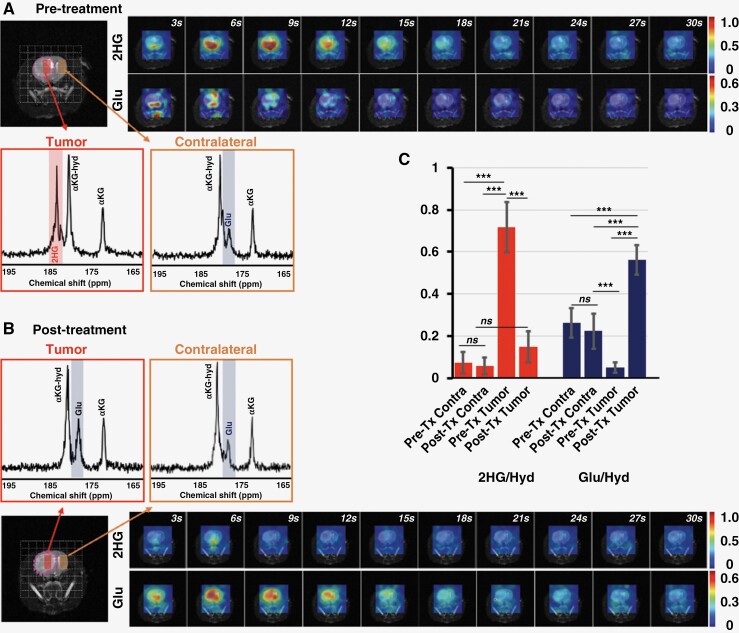
Dynamic heatmaps of 2HG and glutamate obtained from the HP ^13^C EPSI acquisition following [5-^12^C,1-^13^C]α-ketoglutarate injection into rats prior to (A) and following (B) treatment. Inserts illustrate sum spectra over time from tumor and contralateral voxels (red and orange inserts respectively) (C) Quantification of metabolites in tumor and contralateral brain pretreatment and posttreatment (Tx). A *P*-value ≤ .05 was considered statistically significant. *Signifies *P* < .05, **P < .01, ***P < .001, and ns = not significant.

In light of the quality of our spectra and the clearly detectable 2HG peak when injecting HP [5-^12^C,1-^13^C]αKG, we also decided to acquire localized metabolic imaging data using this HP agent. Figure 6 illustrates the dynamic heatmaps of 2HG and glutamate production from HP [5-^12^C,1-^13^C]αKG obtained using our EPSI acquisition sequence prior to (Figure 6A) and following (Figure 6B) treatment at a spatial resolution of 2.5 × 2.5 × 8 mm^3^. 2HG observed within the tumor before treatment was no longer detectable after treatment and glutamate was increased. Summed spectra over time from tumor and contralateral voxels (red and orange inserts in Figure 6A and B) further illustrate the changes in metabolite levels following response to therapy. Quantification of the data (Figure 6C) shows that spectra from contralateral voxels show no changes in normalized metabolite levels (*P-*value = .31 for 2HG, and 0.52 for glutamate). In contrast, posttreatment tumor voxels show significantly lower 2HG (dropping from 0.71 ± 0.12 to 0.15 ± 0.07, *P-*value < .001) and higher glutamate (increasing from 0.05 ± 0.02 to 0.56 ± 0.07, *P-*value < .001) as observed in our cell and animal results described above using a slab acquisition. When assessing the voxel-by-voxel changes in 2HG and glutamate following treatment within the tumor region (*n* = 15), a significant correlation was observed between the drop in 2HG and the increase in glutamate (Supplementary Figure S3, *R*^2^ = 0.49 and *P-*value = .004).

Finally, we also compared the SNRs of our 2 substrates and their products in healthy and tumor-bearing animals (Supplementary Figure S4A and B). We found that the SNRs of our 2 substrates were comparable with each other (SNR = 124 ± 12 for [1-^13^C]αKG and 138 ± 13 for [5-^12^C,1-^13^C]αKG, *P-*value = 0.17) in tumor-bearing animals and were also comparable in normal and tumor-bearing animals (*P-*value = 0.89 for [1-^13^C]αKG and 0.49 for [5-^12^C,1-^13^C]αKG), likely indicating similar substrate delivery. Furthermore, when comparing the SNR of our products between our new sequence and our previously published data (Supplementary Figure S4D),^23,24^ we observed a significant improvement. The SNR of 2HG was about 6 times higher in the current slab study with [1-^13^C]αKG compared to our previous work^24^ in the same tumor model.

## Discussion

Mutations in the IDH enzyme lead to the conversion of αKG into 2HG, the oncometabolite which promotes gliomagenesis while inhibiting anti-tumor immunity.^39^ As a result, inhibiting mutant IDH activity is being considered as a therapeutic approach. Indeed several clinical studies are investigating the utility of these inhibitors, and although mutant IDH has been shown to play its most critical role in tumor initiation,^40^ results to date show some objective responses, including after BAY-1436032 treatment, as well as an increase in cases of stable disease associated with enhanced survival. These results have paved the way for continued clinical trials of these inhibitors either alone or in combination.^7,8,41,42^ But enhanced patient survival following mutant IDH inhibitor treatment has been associated, most commonly, with inhibition in tumor growth particularly when tumor growth is measured volumetrically.^43^ As a result, complementary metabolic imaging methods that can help confirm drug target engagement are essential. In this study, we show that using the commercially available [1-^13^C]αKG or the recently synthesized [5-^12^C,1-^13^C]αKG, combined with a novel pulse sequence, we are able to acquire high-resolution MR spectroscopic imaging data that can readily detect HP αKG-derived 2HG and HP αKG-derived glutamate, as well as their modulation with treatment. Importantly, the drop in HP 2HG production and the increase in HP glutamate production observed in our studies following treatment were also associated with the inhibition of tumor growth and enhanced animal survival.

Previous studies have shown an inverse correlation between tumor response and the ratio of 2HG to glutamate/glutamine detected in the ^1^H MRS spectra of patients^26,44,45^ and similar findings were observed in cells and mouse models.^14,25^ In the current study, we confirmed these observations in a rat model following treatment with BAY-1436032 and demonstrated that treatment that resulted in a significant MRI-detectable inhibition in tumor growth without tumor shrinkage was associated with enhanced animal survival as well as a ^1^H MRS-detectable decrease in 2HG and increase in glutamate. More importantly, however, we also showed that it is possible to use the HP ^13^C approach to rapidly probe the real-time production of 2HG and glutamate from αKG, as well as the treatment-induced modulations in the production of both metabolites. Our metabolic imaging method could therefore provide a noninvasive approach for rapid detection of mutant IDH inhibition in response to treatment. Furthermore, although this study only investigated one astrocytoma model, our method could also be applied to any tumor that harbors the IDH mutation including astrocytoma, oligodendroglioma, and meningioma. It could also be used, as previously described,^23^ for noninvasive assessment of IDH status and tumor detection and characterization, particularly when a tumor cannot be removed or biopsied.

Our prior HP MRS studies using ^13^C labeled αKG to detect 2HG production pointed to initial challenges. Most notably we observed that 2HG is only 0.1 ppm away from the natural abundance peak of [5-^13^C]αKG at 184 ppm making separation of these 2 peaks impossible in the in vivo setting^23,24^ particularly at clinical field strength. Furthermore, the substrate peak was substantially higher than the product peak limiting our dynamic range for the detection of our products. To address these challenges, the study described here leveraged 2 important developments. First, we were able to use the recently described [5-^12^C,1-^13^C]αKG^27^ compound. [5-^12^C,1-^13^C]αKG has a similar T_1_ relaxation time to [1-^13^C]αKG and our SNR findings confirm that, as expected, both compounds penetrate the normal and tumor-bearing brain to the same extent. However, because [5-^12^C,1-^13^C]αKG was synthesized to enrich the C5 position of αKG with ^12^C, this compound no longer generates a resonance at 184 ppm. By removing the peak proximal to 2HG, [5-^12^C,1-^13^C]αKG, therefore, allows us to readily monitor 2HG production at 183.9 ppm without interference. Second, we further optimized the RF pulse. Our pulse excited both possible products of αKG: 2HG, which is the major product in mutant IDH cells, and glutamate, which is the primary product in normal tissue. At the same time, we minimized the excitation of the substrate to conserve its magnetization for subsequent metabolic conversion to the downstream products. Our SPSP pulse delivered an approximate 2° flip angle to the αKG at 172.6 ppm while delivering 78, 78, and 76° flip angles to 2HG, glutamate, and αKG hydrate, respectively. This resulted in a significantly improved SNR of 60 on 2HG compared to an SNR of 11 in our previous slab study.^24^ Furthermore, the minimally excited substrate signal allowed us to monitor the dynamic production of 2HG across 90 s whereas our previous study was able to monitor 2HG for only 12 s. Using the same RF pulse, spectroscopic imaging with EPSI acquisition also showed an excellent SNR for 2HG with a significant improvement over previous findings. In our first study with HP [1-^13^C]αKG^23^ we used less optimal conditions than in this study. In particular, we acquired our data using a clinical scanner rather than a dedicated animal scanner and investigated a tumor model with substantially lower 2HG levels (only ~1 fmol/cell^15^). Nonetheless, using chemical shift imaging with a spatial resolution of 5 × 5 × 20 mm^3^ we were only able to detect 2HG with an SNR of 1.4. Here we were able to detect 2HG with a spatial resolution of 2.5 × 2.5 × 8 mm^3^ and an SNR of 23.

Our enhanced SNR also allowed us to detect the production of glutamate in a normal healthy brain. We cannot rule out the possibility that at least some glutamate is produced elsewhere in the body. However, the relatively lower levels of glutamate observed in the pretreatment tumors compared to normal brains appear consistent with metabolism occurring locally and reflecting the funneling of αKG preferentially to 2HG in tumors and preferentially to glutamate in normal brains.

Our preclinical study has clear limitations. We only investigated one glioma model and one mutant IDH inhibitor. Animal models do not fully recapitulate the behavior of human tumors, and animal response to treatment is not always a good predictor of response in patients. Further studies would therefore be required to confirm our findings. As mentioned above, it is also well-established that the IDH mutation is essential for tumor initiation but subsequent tumor growth can occur independent of the mutation.^29,40^ In such cases, we cannot rule out that inhibition of αKG to 2HG conversion might be uncoupled from inhibition of tumor growth. Our imaging method would then only serve to confirm drug delivery and action at the tumor site but would not be able to predict response. Another important question is whether our imaging method might inform drug resistance. A clinical trial conducted by Hueser et al.^46^ found that BAY-1436032 did not show any potential resistance mechanisms, such as isoform switching or the acquisition of secondary alterations in mutant IDH1, and we are not aware of any preclinical models that would allow us to address this question. Nonetheless, further studies are needed to address this point. Finally, whether sufficient amounts of αKG can be delivered across the BBB in patients remains to be confirmed. Some reports suggest that αKG does not easily permeate the cell membrane.^47^ However, other studies show that brain cells are capable of taking up extracellular αKG,^48^ and, importantly, preliminary studies in normal human volunteers detected the metabolism of αKG to glutamate (Dr. D. Vigneron personal communication).

When considering the translation of this probe to the clinic, αKG is sold in the United States as an over-the-counter nutritional supplement and would not be expected to show toxicity. Furthermore, investigators in the UCSF Hyperpolarized MRI Technology Resource Center recently received FDA investigational new drug approval to test HP αKG as an imaging agent in healthy volunteers and brain tumor patients. Moving forward, our data show that this agent can provide important complementary metabolic imaging information that can help with assessing the impact of treatment in brain tumor patients. Whereas the anatomic MRI-based information can detect treatment-induced inhibition of tumor growth, our metabolic imaging can confirm that the drug has in fact been delivered to the tumor and that the activity of the mutant IDH enzyme has been inhibited in situ. This could potentially help the oncologist make a data-driven, informed decision with regard to further treatment, possibly helping with the management and outcomes of glioma patients.

## Supplementary Material

vdad143_suppl_Supplementary_Tables_S1_Figures_S1-S4
