# Supplementary material for: Monitoring response to a clinically relevant IDH inhibitor in glioma—Hyperpolarized 13C magnetic resonance spectroscopy approaches
Source: Neurooncol Adv. 2023 Nov 2;5(1):vdad143. doi: 10.1093/noajnl/vdad143 (PMC10681661; doi:10.1093/noajnl/vdad143)
Supplement: vdad143_suppl_Supplementary_Tables_S1_Figures_S1-S4 [file vdad143_suppl_supplementary_tables_s1_figures_s1-s4.docx]

**Supplementary Materials**


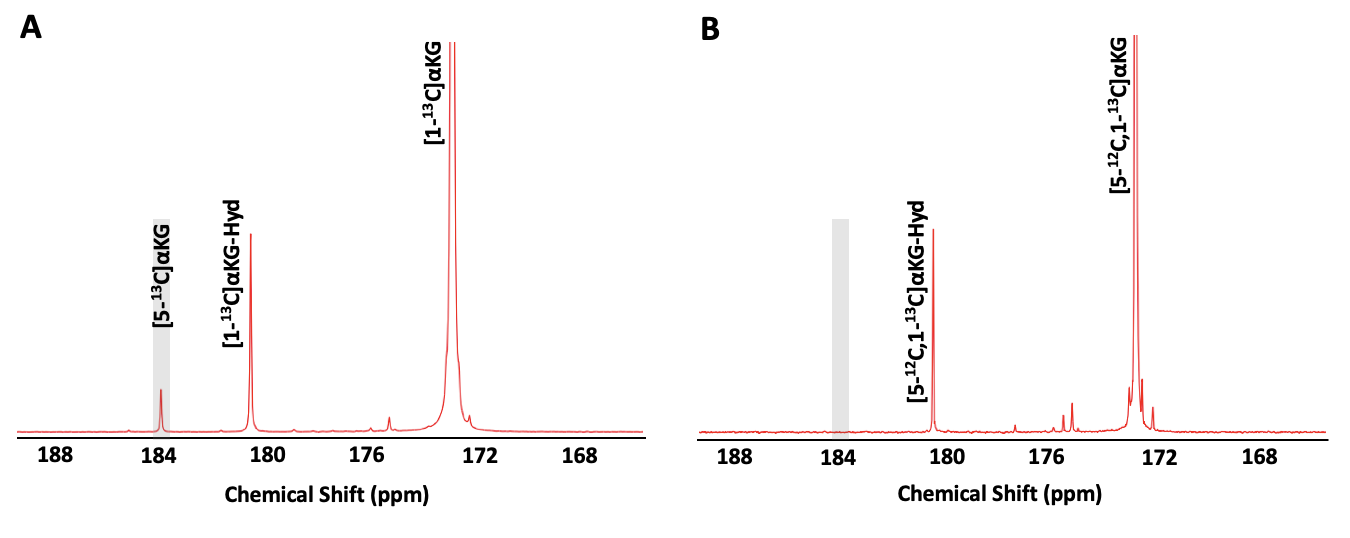


**Figure S1.** ^13^C MR spectra comparing hyperpolarized [1-^13^C]αKG (A) and [5-^12^C,1-^13^C]αKG (B). The natural abundance [5-^13^C]αKG peak at 184 ppm is absent in the [5-^12^C,1-^13^C]αKG spectrum (highlighted in gray).


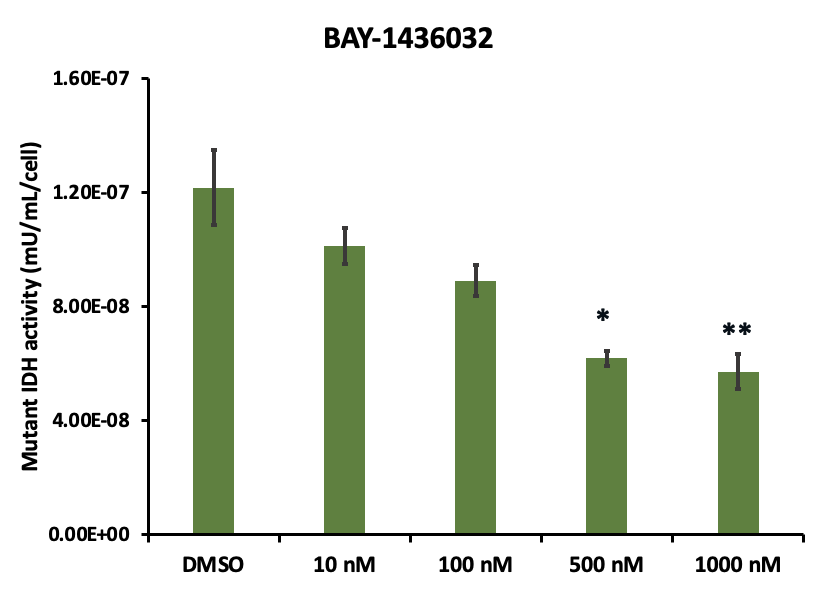


**Figure S2**. IDH1mut enzyme activity in response to BAY-1436032 across different concentrations. This result led to the use of 500 nM as the dose of BAY-1436032 used in to treat the NHAIDH1mut cells.


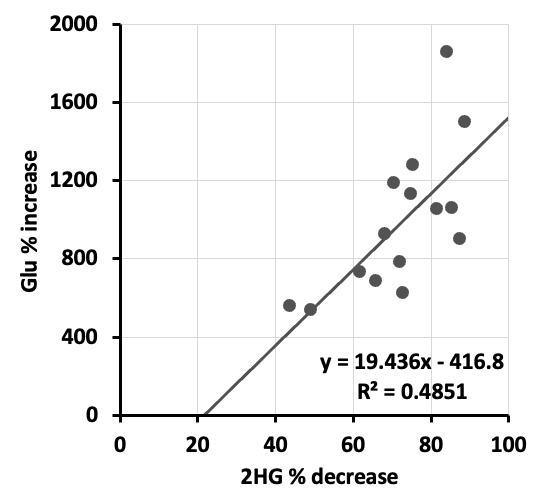


**Figure S3.** Voxel by voxel correlation of 2HG reduction and glutamate increase within the voxels of the tumor region (illustrated in Figure 6) following treatment. Data exhibits a positive correlation with an R square value of 0.49 and a p-value of 0.004.


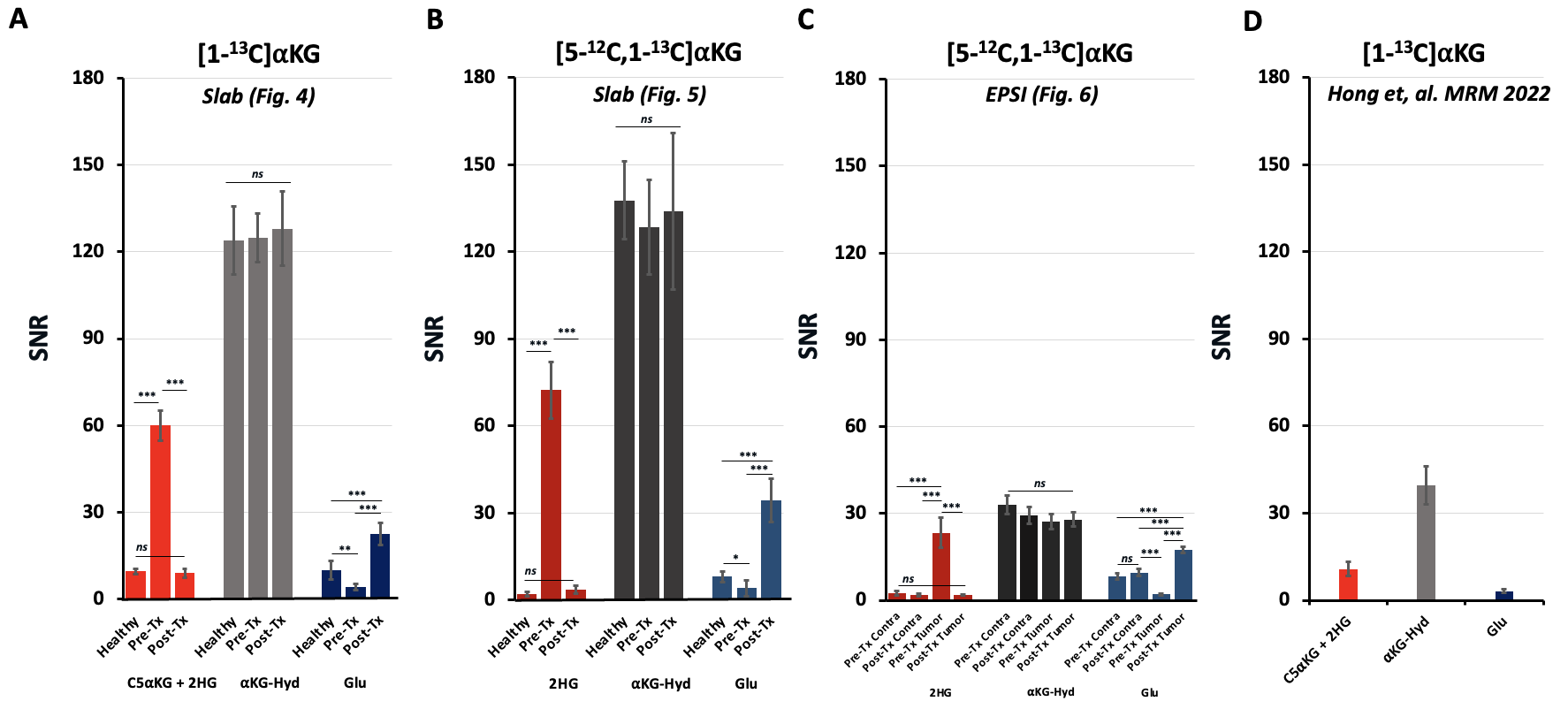


**Figure S4.** SNR comparison for metabolites from (A) slab acquisition with [1-^13^C]αKG, (B) slab acquisition with [5-^12^C,1-^13^C]αKG, and (C) EPSI acquisition with [5-^12^C,1-^13^C]αKG, and (D) previous study with [1-^13^C]αKG (Hong et, al., MRM, 2022, <https://doi.org/10.1002/mrm.29081>)

**Table S1**. T_1_ relaxation times for [1-^13^C]αKG and [5-^12^C,1-^13^C]αKG in our studies and literature at 3 T and 11.6 T

| [1-^13^C]αKG | | | [5-^12^C,1-^13^C]αKG | | |
| --- | --- | --- | --- | --- | --- |
| B_0_ (T) | T (s) | Ref. | B_0_ (T) | T (s) | Ref. |
| 11.6 | 24.3 ± 1.0 | This study | 11.6 | 25.6 ± 2.2 | This study |
| 11.6 | 19 ± 3 | https://doi.org/10.1038/ncomms3429 |  |  |  |
| 3 | 53. 7 ± 1.9 | This study | 3 | 54.0 ± 2.3 | This study |
| 3 | 52 ± 4 | https://doi.org/10.1038/ncomms3429 | 3 | 43.4 ± 0.3 | https://doi.org/10.1002/nbm.4588 |
| 3 | 43.3 ± 0.3 | https://doi.org/10.1021/acschembio.1c00561 | 3 | 38.8 ± 0.3 | https://doi.org/10.1021/acschembio.1c00561 |
